# Perceived enablers and barriers of community engagement for vaccination in India: Using socioecological analysis

**DOI:** 10.1371/journal.pone.0253318

**Published:** 2021-06-25

**Authors:** Tapati Dutta, Jon Agley, Beth E. Meyerson, Priscilla A. Barnes, Catherine Sherwood-Laughlin, Jill Nicholson-Crotty

**Affiliations:** 1 Public Health Department, Fort Lewis College, Durango, CO, United States of America; 2 Department of Applied Health Science, Deputy Director of Research, Prevention Insights, Indiana University School of Public Health-Bloomington, Bloomington, Indiana, United States of America; 3 Research Professor at University of Arizona, Southwest Institute for Research on Women (SIROW), Tucson, AZ, United States of America; 4 Department of Applied Health Science, Indiana University School of Public Health-Bloomington, Bloomington, IN, United States of America; 5 School of Public and Environmental Affairs, Indiana University, Bloomington, IN, United States of America; Public Health England, UNITED KINGDOM

## Abstract

**Background:**

There is high level policy consensus in India that community engagement (CE) improves vaccination uptake and reduces burden of vaccine preventable diseases. However, to date, vaccination studies in the country have not explicitly focused on CE as an outcome in and of itself. Therefore, this study sought to examine the barriers and enablers of community engagement for vaccination in India.

**Methods:**

Employing qualitative methods, twenty-five semi-structured elite interviews among vaccine decisionmakers’ were triangulated with twenty-four national-level vaccine policy documents and researcher field notes (December 2017 to February 2018). Data collected for this study included perceptions and examples of enablers of and barriers to CE for vaccination uptake. Concepts, such as the absence of formal procedures or data collection approaches related to CE, were confirmed during document review, and a final convening to review study results was conducted with study respondents in December 2018 and January 2019 to affirm the general set of findings from this study. The Social Ecological Model (SEM) was used to organize and interpret the study findings.

**Results:**

Although decisionmakers and policy documents generally supported CE, there were more CE barriers than facilitators in the context of vaccination, which were identified at all social-ecological levels. Interviews with vaccine decisionmakers in India revealed complex systemic and structural factors which affect CE for vaccination and are present across each of the SEM levels, from individual to policy. Policy-level enablers included decisionmakers’ political will for CE and policy documents and interviews highlighted social mobilization, whereas barriers were lack of a CE strategy document and a broad understanding of CE by decisionmakers. At the community level, dissemination of Social-behavioral Change Communication (SBCC) materials from the national-level to the states was considered a CE facilitator, while class, and caste-based power relations in the community, lack of family-centric CE strategies, and paternalistic attitude of decisionmakers toward communities (the latter reported by some NGO heads) were considered CE barriers. At the organizational level, partnerships with local organizations were considered CE enablers, while lack of institutionalized support to formalize and incentivize these partnerships highlighted by several decisionmakers, were barriers. At the interpersonal level, SBCC training for healthcare workers, sensitive messaging to communities with low vaccine confidence, and social media messaging were considered CE facilitators. The lack of strategies to manage vaccine related rumors or replicate successful CE interventions during the during the introduction and rollout of new vaccines were perceived as CE barriers by several decisionmakers.

**Conclusion:**

Data obtained for this study highlighted national-level perceptions of the complexities and challenges of CE across the entire SEM, from individual to systemic levels. Future studies should attempt to associate these enablers and barriers with actual CE outcomes, such as participation or community support in vaccine policy-making, CE implementation for specific vaccines and situations (such as disease outbreaks), or frequency of sub-population-based incidents of community resistance and community facilitation to vaccination uptake. There would likely be value in developing a population-based operational definition of CE, with a step-by-step manual on ‘how to do CE.’ The data from this study also indicate the importance of including CE indicators in national datasets and developing a compendium documenting CE best-practices. Doing so would allow more rigorous analysis of the evidence-base for CE for vaccination in India and other countries with similar immunization programs.

## Background

Community engagement (CE) for vaccination has been described as community-based interventions, such as providing communities with adequate information about vaccine benefits and access and supporting all aspects of vaccination services, often targeted at high-risk or vulnerable populations and undertaken by government or implementation organizations [1–3]. While the immediate intended outcome of CE is often to increase community demand for and use of immunization services using downstream methods like home visits by frontline health workers or mass media messaging [4], several studies emphasize CE as an eventual strategy for community-led monitoring and advocacy [5, 6], community equity [7], and ultimately community control for specific health services [8–11]. Notably, multiple international voices have continued to encourage CE to improve vaccination uptake and completion [12], which, in turn, support efforts toward herd immunity and enable countries to achieve sustainable development goals [13, 14].

As one of the 194 member countries that endorsed the World Health Assembly’s Global Vaccine Action Plan framework, India envisions that CE will improve vaccination uptake [15] and increase both communities’ understanding about vaccines and their demand for immunization as their right and collective responsibility [16]. India’s decentralized mechanisms for vaccine delivery are guided by national policy, entirely funded by the Ministry of Health and Family Welfare (MoHFW) and institutionalized through the district-level vaccine plans. Currently, vaccination outreach among the communities is done by multiple frontline healthcare workers, auxiliary nurse midwives (ANMs), Anganwadi workers (AWWs), and Community Health Workers (CHWs), while vaccine sensitization is done by the Accredited Social Health Activists (ASHAs) [17]. The country’s Intensified Mission Indradhanush (initially Mission Indradhanush,—meaning rainbow in Hindi, the Constitutionally approved official language of India—signifying vaccination for seven vaccine preventable diseases: diphtheria, whooping cough, tetanus, polio, tuberculosis, measles and Hepatitis B) set a goal to completely vaccinate 90% of vaccine-eligible communities in the country, especially those which are mobile or isolated, have low vaccine demand, or have high vaccine resistance [18]. However, the latest survey by the Ministry of Health and Family Welfare found that India’s vaccination rate was 62% (NFHS-4, 2015–16), a figure that stands alongside caste, class, and gender-based differences in vaccination outcomes [19, 20].

Studies have identified myriad factors related to vaccination refusal and hesitancy, including refusal for religious, social, and philosophical reasons [21, 22], lack of trust in vaccine providers and others [23], and fear of vaccines and adverse outcomes following immunization [24]. At the same time, Weigmann (‎2017) [25] suggests that mandatory vaccination is an infringement on a community’s freedom of choice. One can observe lower vaccination rates in cases where a top-down immunization structure is not likely an effective approach but is nonetheless utilized [26]. For example, CE interventions may be de-prioritized in favor of ‘scare tactics’ [27] without opportunities for community involvement in planning, monitoring, and surveillance activities [28, 29]. Salathé and Bonhoeffer (2008) [30] suggest this lack of CE may reduce trust in vaccines and in vaccine decisionmakers in the eyes of communities [31, 32]. Other scholars have attributed vaccine hesitancy, refusal, and even backlash to questionable or non-existent CE efforts [33, 34].

In India, as elsewhere, vaccine decisionmakers or ‘elites’ [35], as well as formal vaccine policy documents endorsed by the MoHFW, Government of India, jointly drive national vaccination policy and programs. Thus, these sources of information can be used to elucidate the complexity and comparative effectiveness of CE implementation regarding vaccination [36, 37]. While policy documents can be relatively easy to access because they are publicly available, in many cases, intensive elite interviews may be more difficult to conduct. Literature on elite interviewing notes that ‘elites’ are infrequently interviewed because such people are hard to reach, surrounded by gatekeepers, and have the power to protect themselves from intrusion and criticism [38–40]. Despite those barriers, this study presents qualitative analyses derived from both policy documents and more than two dozen elite interviews conducted to identify perceived CE enablers and barriers for vaccination in India. This information closes an evidentiary gap and enhances current literature on vaccination and CE in India, which is mostly based on views of communities or local stakeholders [41, 42].

## Methods

### Data sources and inclusion criteria

#### Elite interviews

The study employed a multi-method, sequential, qualitative approach to identify the key enablers and barriers to CE for vaccination uptake in India as perceived by vaccine decisionmakers and/or reflected in the vaccine policy documents of the country. The data sources consisted of (1) a set of semi-structured intensive elite interviews with vaccine decisionmakers in India, supplemented by follow-up convening meetings with these participants to clarify particular aspects discussed during the interviews; and (2) national-level vaccine policy documents in India. Ethical approval was received from the Indiana University’s Institutional Review Board (Protocol number 1710654732A001) and informed consent was obtained via email from all individual study participants.

For this study, ‘elites’ were defined as vaccine decisionmakers, in positions of authority for at least seven years, who were responsible for one or more of the following: formulating vaccine policies and programs in India, signing off on the introduction and roll out of vaccines under the Universal Immunisation Programme, and/or carrying out vaccine clinical trials between 2010 until the present. This conceptualization of ‘elites’ is consistent with prior research on this approach: people who are not necessarily of high social, economic, or political standing, but rather are defined by who they are or the position they occupy (e.g., titles or positions) in a particular domain area [43].

For this paper, ‘vaccine policy documents’ were defined as any publicly available (digital or hard copy), vaccination-related strategy and/or operational guideline, that were jointly published by the MoHFW and the National Health Mission, India’s flagship health-sector programs to revitalize rural and urban health, since 2010.

The lead author and researcher [TD] identified the key roles and positions of authority of vaccine decisionmakers and elites, then used public sources of data to determine who currently held each of those positions, yielding an initial purposive sample of 30 individuals. It was anticipated that this approach would be most likely to achieve information saturation by virtue of its attempt to coalesce data from all eligible elites rather than a subsample. Thereafter through personal networking, and informal outreach by TD, twenty-eight participants were contacted. Approaching many of these individuals to request participation was made possible because of TD’s earlier work on CE with some of these vaccine decisionmakers, and thus a level of professional knowledgeability, acquaintance and trust between the both [44]. First, an email was sent to all the 28 decisionmakers in December 2017 explaining the study information and requesting an invitation for an interview (a sample of the email highlighting study purpose and requesting study participations can be found in S1 File). In case of non-response from potential study participants or their offices, follow up emails were sent in early January 2018, after around ten working days. In most cases, once the decisionmaker consented via email to participate in the study, follow up phone calls and emails were sent to their offices requesting an interview date between 12–15 days after the initial invitation. Of the 28 elites for whom contact information was located, three were excluded from the list because one did not have a physical office in India, one could not be contacted, and one person initially agreed but could not participate in the interview because he was busy with new vaccine introduction in the country. However, his counterpart working in same Government institution participated in the study, which allowed us to address theoretical saturation.

**Fig 1:**
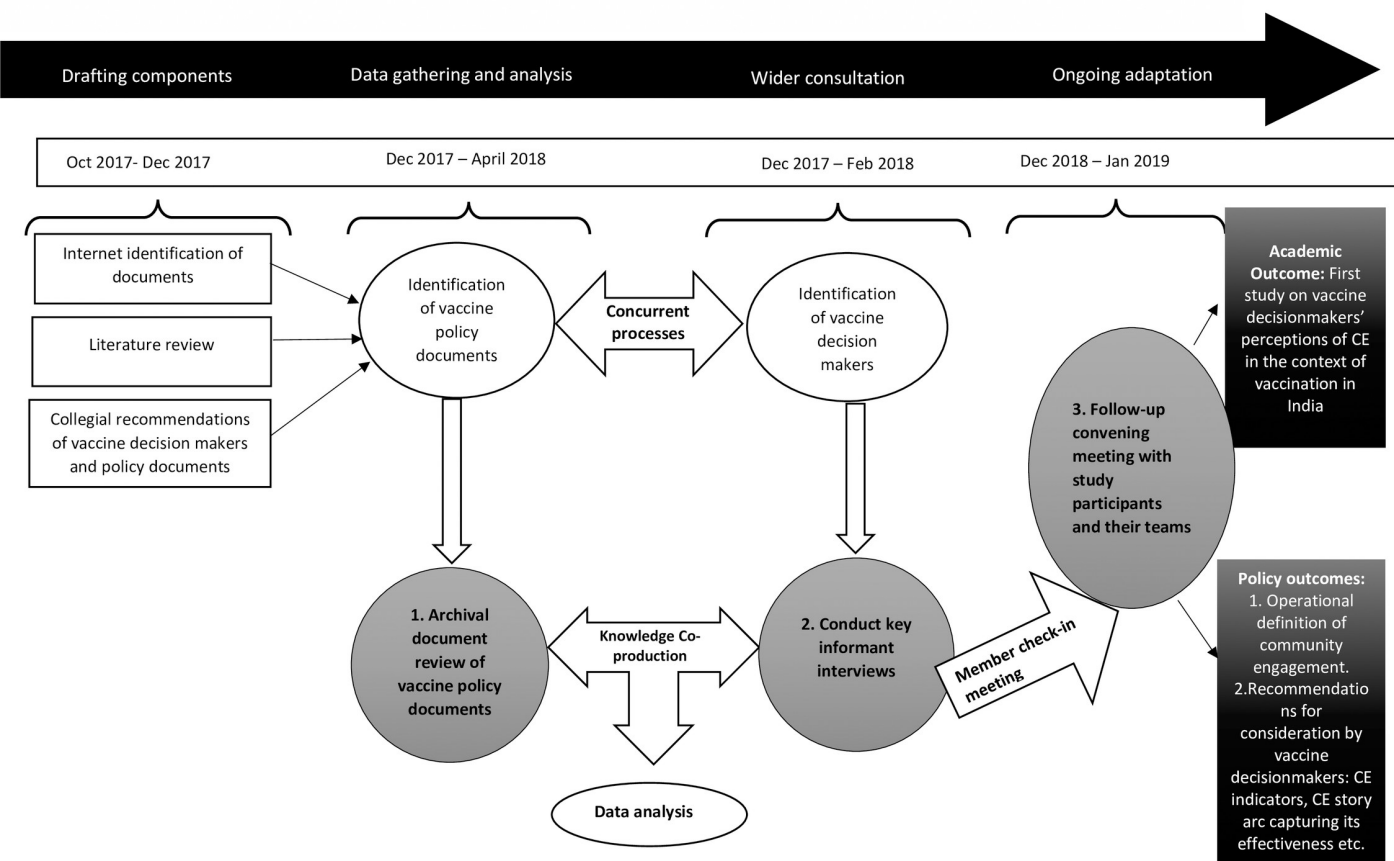
Visual depiction of sequential, and multi-staged qualitative study design to examine community engagement (CE) for vaccination in India, 2018.

For most respondents, two emails followed by a call yielded an affirmative response. However, for representatives at the Ministry, an initial email from TD, followed by an introductory/reference phone call by another contemporary in the Government/donor/technical organization, was required to access the potential interview participants and recruit them. Twenty-five vaccine decisionmakers participated in the one-on-one interviews. Details of the inclusion process of the study participants and vaccine policy documents is explained in Fig 1.

Of the 25 elites who participated in the interviews, two held secretary-level positions, the highest office at the Ministry of Health Family Welfare for the Universal Immunization Program. Seven were from technical and research institutes under the aegis of Ministry and were responsible for vaccine policy, operational guidelines, program formulation and approval, vaccine supply, cold-chain management, and certifying ethical conduct of clinical trials. Three participants from three different UN organizations oversaw vaccine surveillance, uptake through social mobilization, and training of local stakeholders. Three immunization heads in donor organizations led strategic partnerships along with the Ministry of Health Family Welfare to ensure vaccination funding. Four technical heads from three multi-country, multi-partner projects, and six country leads of non-governmental organizations (NGOs) collaborated with the government, donors, and technical partners in ensuring achieving the UIP goals or engaged with communities to recruit participants and conducted vaccine clinical trials.

#### Policy documents

A scoping review of the overall content of the vaccine policy documents was conducted due to the diversity of the archival documents, broad scope of CE, limited time, and lack of any earlier national-level review on this topic [45]. Boolean internet searches conducted from October to December 2017 yielded 20 policy documents for review. The objective was to identify pertinent vaccine policy documents using the search string: ‘vaccine’ AND ‘policy’ OR ‘guideline’ AND ‘India.’ Inclusion criteria for the vaccine policy documents were: national-level vaccine policies, vaccine strategy documents, vaccine specific guideline documents, frequently asked question (FAQ) documents on vaccines for different stakeholders such as parents or healthcare providers, and documents on adverse event following immunization (AEFI) in India. These documents addressed immunization goals and provided information to stakeholders and communities about the public health benefits about vaccination. Four additional documents were suggested by study participants, leading to twenty-four documents which were finally included in the study. These documents were jointly published by the MoHFW and the National Health Mission (NHM), country offices of the World Health Organization (WHO), United Nations International Children’s Emergency Fund (UNICEF), and/or the CORE Group Polio Project (CGPP), between 2010 and early 2018, when the review was being conducted. Accordingly, these vaccine policy documents were either available on the website of the Ministry, or the National Technical Advisory Group on Immunization (NTAGI), or the Immunization Technical Support Unit (ITSU). While all the documents were available both in English (the official/professional language mostly practiced in India) and in Hindi (the Constitutionally approved official language of India), only the English versions of these vaccine policy documents were included and reviewed in this study.

Of the 24 vaccine policy documents, 15 (62.5%) were published between 2015 and 2018 and were vaccine-specific operational guidelines introduced during those years by the Ministry. Depending on their content, the documents were classified into five categories: policy and program review documents (*n* = 3, 12.5%), vaccine and program-specific operational guidelines (*n* = 7, 29.1%), FAQ booklets for communities and community-level stakeholders (*n* = 3, 12.5%), AEFI documents (*n* = 3, 12.5%), and social and behavioral change communication (SBCC) and social mobilization related documents (*n* = 8, 33.3%). An overview of the vaccine decisionmakers (N = 25) and policy documents (N = 24) is provided in Table 1.

**Table 1:** Overview of vaccine decisionmakers and policy documents in India selected for this study, 2018.

Study participant (N = 25)	Study participant’s organizational classification *Govt*. *of India*, *MoHFW = GoI Ministry*, *State-level nodal institution of the Ministry = GoI*, *Ministry (S)*, *Technical & Advisory consortium under the aegis of the Ministry = GoI*, *Technical Org*, *HQ in a developed country with country office in India = Intl*. *NGO in India*, *Principal financial recipient from a foreign donor and programmatic ownership of Govt*. *of India = NGOs/Principal recipient with an India office)*	Study participant’s community engagement role for vaccination *(Establish regulations = Regulatory*, *Carry out surveillance/research = Surveillance/research*, *Provide funding = Financial support*, *Develop policy guidelines/technical support = Technical support*, *Develop communication strategies and materials = Communication strategies*, *Implement nationally sanctioned policies and programs = Policy and program implementation)*	Vaccine Policy Document (N = 24)	Title of the Document & Year of publication	Publishing authority	Document type *Policy and Program review documents = Policy&Prog; Vaccine and program specific operational guidelines = Vax Opr Guide; FAQ booklets for stakeholders = FAQ booklets; AEFI related documents = AEFI documents; Communication and Social mobilization related documents = CE & SBCC docs*
1.	GoI, Ministry	Regulatory	1.	National Vaccine Policy (2011)	MoHFW, GoI	Policy&Prog
2.	GoI, Ministry	Regulatory	2.	Midterm Review Multi-Year Strategic Plan 2013–17 (2016)	MoHFW, & NHM, GoI	Policy&Prog
3.	*GoI*, *Ministry (S)*	Policy and program implementation	3.	Universal Immunization Program Reaching Every Child 2013–17	MoHFW, GoI	Policy&Prog
4.	*GoI*, *Ministry (S)*	Policy and program implementation	4.	Mission Indradhanush, Operational Guidelines (2015)	NHM, GoI	Vax Opr Guide
5.	*GoI*, *Ministry (S*	Policy and program implementation	5.	Intensified Mission Indradhanush, Operational Guidelines (2018)	MoHFW, & NHM, GoI	Vax Opr Guide
6.	GoI, Technical Org	Technical support	6.	Operational Guide Japanese Encephalitis Vaccination in India (2012)	NRHM, GoI	Vax Opr Guide
7.	GoI, Ministry	Policy and program implementation	7.	Introduction of Measles–Rubella Vaccines, Campaign and Routine Immunization (2017)	MoHFW, & NHM, GoI	Vax Opr Guide
8.	GoI, Ministry	Regulatory	8.	Operational Guidelines for Introduction of Inactivated Poliovirus Vaccine (2015)	MoHFW, & NHM, GoI	Vax Opr Guide
9.	GoI, Ministry	Regulatory	9.	Operational Guidelines Introduction of Rotavirus Vaccine in the Universal Immunization Programme in India (2015)	MoHFW, & NHM, GoI	Vax Opr Guide
10.	Intl. NGO in India	Regulatory and Surveillance/research	10.	Operational Guidelines, Pentavalent Introduction (DPT+HepB+Hib) (2014)	MoHFW, & NHM, GoI	Vax Opr Guide
11.	Intl. NGO in India	Technical support and Communication strategies	11.	FAQ on Immunization for Parents & Caregivers (2017)	MoHFW, & NHM, GoI	FAQ booklets
12.	Intl. NGO in India	Regulatory	12.	FAQ on Immunization, for Health Workers & Other Front-line Functionaries (2017)	MoHFW, & NHM, GoI	FAQ booklets
13.	Intl. NGO in India	Financial support & Technical support	13.	FAQ on Immunization for Religious Leaders, Media Persons, CSOs, Influencers & Other Stakeholders (2017)	MoHFW, & NHM, GoI	FAQ booklets
14.	Intl. NGO in India	Financial support & Technical support	14.	AEFI Media Communication Protocol	ITSU and NHM, GoI	AEFI documents
15.	Intl. NGO in India	Financial support	15.	AEFI Surveillance and Response Operational Guidelines (2015)	MoHFW, and NHM, GoI	AEFI documents
16.	NGO/Principal recipient	Communication strategies & Policy and program implementation	16.	National Quality Assurance Standards for AEFI Surveillance Program (2016)	MoHFW, and NHM, GoI	AEFI documents
17.	NGO/Principal recipient	Communication strategies & Policy and program implementation	17.	Social Mobilization, Lessons from the Core Group Polio Project in India (2012)	USAID and CORE Group	CE & SBCC docs
18.	NGO/Principal recipient	Communication strategies & Policy and program implementation	18.	Intensification of Routine Immunization Communication Operational and Technical Guideline (2012)	NRHM, and MoHFW, GoI	CE & SBCC docs
19.	NGO/Principal recipient	Communication strategies & Policy and program implementation	19.	Evaluation of Social Mobilization Network, Final Report Main Section (2014)	UNICEF	CE & SBCC docs
20.	NGO, India office	Surveillance/research & Technical support	20.	GAVI UNICEF Alliance Partnership Document with India (2015)	GAVI	CE & SBCC docs
21.	NGO, India office	Surveillance/research & Technical support	21.	CORE India Communication Strategy (2017–2022)	CORE India	CE & SBCC docs
22.	Intl. NGO in India	Regulatory & Surveillance/research	22.	Standard Operating Procedures for engaging with youth institutions for social mobilization for IMI and RI (2018)	GoI, MoHFW, Rotary International & National Polio Plus Committee	CE & SBCC docs
23.	Intl. NGO in India	Surveillance/research & Technical support	23.	Communication Guidelines for Building Vaccine Confidence around AEFI (2013)	NRHM, and MoHFW, GoI	CE & SBCC docs
24.	Intl. NGO in India	Policy and program implementation	24.	Communication Guidelines for Building Vaccine Confidence around AEFI (2016)	MoHFW, GOI, WHO, UNICEF and Rotary International India National Polio Plus Committee	CE & SBCC docs
25.	Intl. NGO in India	Policy and program implementation				

FAQ: Frequently Asked Questions, ITSU: Immunization Technical Support Unit, GoI: Government of India, MoHFW: Ministry of Health and Family Welfare, NHM: National Health Mission, NRHM: National Rural Health Mission, MoHFW, GoI, GAVI: The vaccine Alliance, UNICEF: United Nations International Children’s Emergency Fund, USAID: United States Agency for International Development, WHO: World Health Organization

### Procedure

All the elite interviews were conducted by TD, in-person, between December 2017 and February 2018, in the offices of the decisionmakers that were either located in New Delhi, the capital city of India, or in the National Capital Region, which is around New Delhi.

For the elite interviews, an interview guide was developed after referring to two studies highlighting decisionmakers perspectives of vaccine introduction and rollout in Bangladesh and Rwanda. These two studies were used as methodological touchpoints because, similar to the Indian context, the Rwandan and Bangladeshi decisionmakers identified upstream drivers like research findings on vaccine-preventable diseases, participation of technical committees and professional bodies, political issues relating to disease outbreaks, pressure from international development partners, community-based tailored vaccination delivery strategies, and engaging community health workers for effective CE for vaccine introduction and rollout [46, 47].

The semi-structured interview guide addressed the following areas: (1) the respective institution’s history with community engagement to improve vaccination uptake in India since 2010, (2) decisionmakers’ conceptualization of CE in the context of vaccination, (3) experiences and examples of the decisionmakers supporting/facilitating community engagement for vaccination [48]; (4) perceptions and examples of enablers to promote community engagement for vaccination, (5) perceptions and examples of barriers to community engagement for vaccination. The interview guide and areas of content analysis were guided by Weert’s and Sandman’s work on the civic-oriented agenda of leadership in institutions of higher education with questions like (1) ‘Does the executive leadership of the institution explicitly promote community engagement as a priority?’ and (2) ‘Is community engagement defined and planned for in the strategic plans of the institution?’ [49]

The interview guide was inductively refined based on emerging themes throughout the interview processes. All the interviews were conducted in English, audio recorded with consent of the interviewees (none refused consent) and lasted for approximately for an hour.

To document non-verbal data like body language of the interviewee, intermittent phone calls, or calls for emergency meetings, which are very real aspects of ‘elites’ work profile, field notes were taken in parallel to the audio recording. No financial incentive was provided to study participants. After each interview summary field notes were taken to allow emerging insights to be included in the subsequent interviews. For example, in the first two interviews, CE barriers and the role of the media, which perceived to be hindering the introduction of the HPV vaccines at that time, came up, and thus were explored in the interviews thereinafter.

The scoping review of the content of the vaccine policy documents was completed in conjunction with interviews and field notes of these interviews. The documentary evidence, combined with data from interviews and fieldnotes, allowed the researcher to counter threats to trustworthiness such as reactivity, minimized respondent bias, and established credibility, while identifying CE themes and CE-related enablers and barriers. The Socio-Ecological Model (SEM) conceptual framework was adapted and used for the coding, categorizing codes into policy-level factors, community-level factors, organizational-level factors, interpersonal-level factors, and individual-level factors. This is not to suggest that data were collected from each of those levels, but rather that perceptions of the national-level elites were categorized and coded to each of the SEM levels mentioned above.

After preliminary analyses, and in order to confirm the elite interview findings and the completeness of the data, TD convened a follow-up meeting with all the study respondents as a group in December 2018 to present and verify study observations. Some study respondents invited their work teams to participate in this meeting and three of the respondents who could not be present during the meeting met TD separately in one-on-one meetings in January 2019. There were no appreciable changes to the findings based on the feedback. Visual depiction of this multi-staged and concurrent qualitative study design is explained in Fig 1.

### Conceptual framework

The SEM was utilized as an organizing framework for data analysis because it describes factors at multiple levels, including policy, community, organizational, interpersonal and individual. The selection of the SEM reflects the multi-level nature of vaccine interventions, and likely attempts at or conceptualization of CE. For example, policy-level factors include policies and regulations affecting communities and the institutions, community-level factors are often functions of the relationship among different institutions within communities, organizational-level factors constitute institutional organization and management, the interpersonal-level factors include interactions of individuals with families, peers, neighbors, and healthcare workers and the individual-level factors include vaccine related beliefs, values, and other individual factors [50–52]. Although SEM has been widely accepted, and used in a few vaccine studies [53, 54], and vaccination and vaccine clinical trials have studied community’s, program personnel’s and healthcare providers perceptions on enablers and inhibitors of CE [55, 56], no study has used SEM to characterize and understand barriers and enablers to CE for vaccination in India, from vaccine decisionmakers’ perspective. Adapting the SEM framework, this study defined policy-level factors as policies and regulations to advance CE for vaccination, the community-level factors were often the decentralized dissemination of SBCC materials among different communities, the organizational-level factors constituted institutional organization and partnerships with the government to advance CE activities, the interpersonal-level factors include interactions of vaccine decisionmakers with priority populations and communities, and the individual-level factors include vaccine decisionmakers individual actions if any, to promote CE for vaccinations.

### Coding and data analysis

The interviews were professionally transcribed, with subsequent integrity checking undertaken for accuracy. A deductive, thematic approach informed by the SEM was used to identify recurring and emerging themes. This was followed by a clustering of data into nodes and sub-nodes [57, 58]. *A priori* coding included barriers/facilitators of CE, which were then classified into each social-ecological level. Thereafter, line, sentence, and paragraph segments of transcribed interviews, policy documents, and field notes were reviewed repeatedly to identify barriers to and facilitators of CE. Two additional and independent researchers coded a sample of five interviews and two policy documents to assure study rigor. Repeated coding conferences were held to identify and negotiate coding discrepancies. Categories with coding differences were addressed and re-defined through iterations until 90% consensus was achieved [45]. The preliminary codes helped coders to integrate already well-known concepts from extant literature. Care was taken not to compulsively fit data into predefined codes, though a ‘start list’ [59] allowed new inquiries to benefit from and build on previous insights.

The identified concepts were reintegrated into themes, which provided the structure for the study results. This led to a form of pattern recognition, with emerging themes/nodes becoming the categories for analysis [60]. In total we identified 10 underlying factors. All the 25 interviews and 24 documents were re-coded using this finalized instrument in Nvivo 12 (QSR International, Melbourne, Australia).

## Results

The analyses identified more CE barriers than facilitators at all the social-ecological levels. Study outcomes are summarized in Table 2 and described by category subsequently.

**Table 2:** Summary of Community Engagement (CE) enablers and barriers for vaccination in India reported by national-level vaccine decisionmakers and policy documents, by levels of Socio-Ecological Model (SEM), 2018.

SEM Levels	CE facilitators6	CE barriers
Policy-level factors	• Evidence of political-will for CE.	• Predominantly social mobilization approach, rather than CE
	• Evidence of direct communication between decision makers and communities addressing vaccination related inquiries	• Adhoc CE interventions during AEFIs or any other emergency
		• Lack of any CE indicator
		• Lack of CE policy/strategy document
		Lack of village level communication plan
Community-level factors	• Publication and dissemination of targeted SBCC materials	• Evidence of class, caste, gender, rural versus urban related power-structures in communities
		• Skewed power relations between communities and health staff or vaccinating authorities
		• Top-down power relations between NGOs and Government/donors
		• Lack of sub-population specific SBCC materials
		• Lack of family-centric strategies to promote consultative household level vaccination decision making
		• Lack of evidence in policy documents highlighting power relations between stakeholders
Organizational-level factors	• Evidence of formal partnerships between national and local stakeholders (religious leaders, clubs, women’s groups)	• Lack of formalization of partnerships between national and local stakeholders (religious leaders, clubs, women’s groups)
		• Lack of evidence of partnership aiming to strengthening CE
		• Lack of quality investment in understanding community sentiment and tailoring trainings and SBCC materials accordingly
		• Lack of consistent strategic planning or policy guideline for contested vaccines
Interpersonal-level factors	• Evidence of evolution of sensitive messaging in vaccination related IPC and SBCC documents	• Lack of any mention of social-media proliferation in policy documents
	• MI logo	• FAQ documents, irrespective of the target group had the same language and presentation
	• Evidence of utilization of social media as much as traditional media for SBCC	• No evidence of replicating SBCC interventions during Polio campaigns for new vaccines
	• Tailored SBCC with men and mothers in law, considering the patriarchal setting in India	• Complex language in AEFI documents
		• Decision makers did not take ownership of contested vaccines or any AEFIs
		• Lack of rumor management strategies
Individual-level factors		• Non vaccination or lack of CE was mostly positioned as the community’s fault
		• Use of physical power to manipulate vaccination decisions

### Policy-level CE facilitators

Policy-level CE enablers included increasing political will for CE and elites’ intent to support bottom-up CE measures to facilitate community interactions during vaccine introduction and roll-out. For example, in the context of school-based HPV vaccination in Punjab State, India, one interviewee noted:

“*First the schools were not so involved*, *but then Karnataka State showed us the way* (the ‘how’) *and we improved our strategy* (to engage with the communities) *so that in the next phase the school*, *teachers and students are involved in a big way*. *We did mid-term evaluation*. *That is why I am saying that involvement of the community is very important*.*”*

More than half of the participants noted that communication directly between the decisionmakers and communities facilitated CE, such as:

“*Because our email ids are there on the website*, *emails* (stating that) *to the Minister or the Prime Minister’s Office saying*, *“my child has not been approached for full immunization”*, *or “this vaccine is not available in my community”… and then we reply*.*”*

Some participants described the regular home visits during the National Polio Surveillance Program as an effective CE strategy. That campaign was initiated in 1995 to ensure polio eradication through house-to-house poliovirus vaccine delivery.

“*When we went to a village*, *we sat with them* (villagers) *and asked if they wanted to talk with us*. *We would ask*, *why the children were not getting immunized*. *Then they asked what the harm is if they did not get immunized*? *Or the advantage of getting immunized*? *So*, *we would sit down and answer their questions*.*”*

### Policy-level CE barriers

Most participants acknowledged decisionmakers’ complacency with a social mobilization approach to “*doing real CE*.” These participants identified a dearth of “*institutionalization of CE*,” which reinforced the distance between the decisionmakers and communities. Highlighting the lack of empowered communication, most participants critiqued the community and home visits as *“ad-hoc*,*”* rather than systematic CE approaches.

“*Communities need to be given a platform where they can share their viewpoints*. *There is no direct channel of communication between the community and the policy maker*. *If it were there*, *these leaders and policymakers would need to think twice*. *These public responses are thought of only when there is an adverse event*, *like in Tamil Nadu State recently for M-R vaccine campaign*.*”*

Two participants commented on the lack of any CE indicator for vaccination uptake in national surveys such as the National Family Health Surveys, or any studies examining CE’s effectiveness as barriers to strategically defining, planning, implementing, and assessing CE.

“*The demand side barriers are very special*, *and they require engagement and understanding*. *It cannot be done with one coverage evaluation survey done six years ago which says 40% of this* (lack of immunization) *is because of demand*. *I cannot unpack that*. *I can do nothing with that information*.*”*

More than half of the participants reported the lack of any dedicated policy document on CE and the absence of village-level communication plans. Documents, such as Communications for Development, Strategy for Hard to Reach Populations, and CORE Communication Strategy, were cited as the nearest to any CE strategy. However, there was no evidence of any community involvement in formulation of these documents or in translating them to regional languages, local dialects or mother-tongues.

### Community-level CE facilitators

Publication and decentralized dissemination of targeted SBCC materials from the national-level to the states, districts, and local levels was identified as the only community-level CE enabler because these documents facilitated community-level interactions among high vaccination resistance and low vaccine coverage communities. Participants described a variety of reminders/prompts and interpersonal messaging through mobiles, WhatsApp, door-to-door canvassing, and strategic use of itinerant megaphones. These were carried out by frontline health care providers, local vaccine champions (community people who share their personal stories for getting themselves or their children vaccinated, and in the process help increase vaccination-related trust and confidence among their friends, families, neighbors and community), stakeholders representing minority religious groups, migrant laborers like brick-kiln workers, and Scheduled Castes and Scheduled Tribes—designated as historically disadvantaged and systematically deprived communities by the Constitution of India.

Review of vaccine policy documents revealed use of group-based dialogues and vaccine champions to build peer-pressure and mobilize vaccine hesitant and resistant communities for pro-vaccination decisions. Examples were: *‘FAQ on Immunization for Religious Leaders*, *Media Persons*, *CSOs*, *Influencers & Other Stakeholders’* and sections in the ‘*Social Mobilization’* document like *“Devote time to the selection*, *training*, *and support of community-based outreach workers*”; *“Advance the participation of women as social mobilizers*, *vaccinators*, *surveillance officers*, *and leaders in polio eradication efforts”*; and *“Involve children in campaigns to help counter ‘campaign fatigue*.”

### Community-level CE barriers

Three decisionmakers described incidents which apparently impeded communities’ vaccination decisions. These were described as examples of systemic discrimination and ranged from class- and caste-based power-relations within communities, to communities’ depleted trust in ‘government-systems’. For example:

1. *A vaccination camp was organized closer to the higher caste person’s house leading to lesser turnout of the lower caste communities to this camp*. [Caste-based discrimination]
2. (Earlier) *A case of adulterated yellow potable water from a Government installed hand-pump when [the] adjoining communities became suspicious to any Government initiatives*, *including vaccinations*. [Government-community divide and lack of trust]

Some participants also described the community’s subjugated positioning and the Government’s paternalistic engagement for vaccinations as a barrier to CE:

*“I see an obsession with immunization*, *which may not necessarily mean community engagement*, *unfortunately*. *It may mean loud noise*. *It may mean perverse incentives*. *We will not give you ration* (food provided at a subsidized rate by the Government to poor families) *because you did not get your immunization*.*”*

*“I think both the Government and other stakeholders are talking down to communities*. *That needs to change*. *In that arena*, *I do not think we have progressed much in the last 10 years*. *We are still not telling the community what these* (vaccines) *are about*. *We are only telling them that they need to get them*.*”*

Another power-related nuance was expressed by NGO heads, who felt that the *‘Government is trying to clip their* (NGO’s) *wings*.*’* They recommended an *‘integrated approach*,’ but did not have ideas for how to implement or define it. On the other hand, policy documents had altruistic recommendations without any articulation of power-equations, or any recommendations for cultivating an enabling environment for CE quality approaches. For example:

“*Enabling processes for rapid decision making to allow building alliances and partnerships*, *both national and global*, *and for support to agencies for diffusion of the technologies into the social systems*, *should be in place*.*”* (Source: National Vaccine Policy, Ministry of Health and Family Welfare, 2011).

Furthermore, the language, especially in the Adverse Event Following Immunization (AEFI) documents, appeared to have conceptualized communities merely as vaccine beneficiaries rather than articulating any empowered role in vaccine policy making or program implementation. These documents neither attempted to demystify AEFI concepts for communities nor combat communities’ misplaced concerns over vaccines. For example:

“….*engage and convince communities after India achieving ‘polio-free status’ especially mothers and caregivers on the importance of vaccinating their children again and again for and during polio campaigns*.*”* (Source: Intensification of Routine Immunization: Communicational Operational and Technical Guideline, MoHFW, 2012).

Power-equation-based contentious issues and contested vaccines, like HPV, seemed to be a mutual function of each other. While answering questions about HPV vaccinations in the country, five participants credited the frontline healthcare workers for successful introduction of HPV vaccines in few states of the country while several exhibited discomfort with sharing any plans for HPV vaccine introduction in the near future. One person changed the topic, and two participants mentioned that they did not work on HPV vaccines after demonstration projects were stalled in 2010, and thus they would not be able to answer. Further, the lack of operational guidelines for HPV vaccines can itself be considered CE barrier.

### Organizational-level CE facilitators

Participants and policy documents highlighted bilateral partnerships with organizations such as GAVI, The Vaccine Alliance, WHO, and UNICEF, as well as inter-sectoral technical groups.

“…*when we talk of community engagement there are two platforms*, *one is State Task Force on Immunization and another is the District Task Force on Immunization*, *who have regular meetings*.*”*

Participants also expressed their reliance on local youth organizations, namely the National Cadet Corps, Nehru Yuva Kendra, National Service Scheme, and Rotary International, and credited them for undertaking social mobilization activities to facilitate achieving MI’s and later IMI’s vaccination target. These activities consisted of infotainment programs and involvement in the district task force meetings. Only one document, *‘SOP on Engagement of Youth Organizations and Rotary Club for Immunization*,*’* was identified.

The National Vaccine Policy acknowledged the virtue of partnerships to advance vaccine development and delivery roles rather than enhancing any CE for vaccination:

“..*several examples where product development have taken the Public Private Partnership route and have resulted in shortening of the time frame for vaccine development*, *such as the Meningococcal Meningitis Vaccine Initiative*, *where the product was produced in India with multiple partners*, *met international standards in quality*, *was exported to and used in Africa*. *The model has been instrumental in indigenously 116E Rotavirus vaccine being developed with effective collaboration between Indian & US academia*, *and Indian vaccine industry in partnership with PATH*.*”* (Source: National Vaccine Policy, Ministry of Health and Family Welfare, 2011).

### Organizational-level CE barriers

Participants emphasized that a lack of Memoranda of Understanding, monetary incentives, or salaries for local youth organizations, women’s self-help groups, community-based organizations, and faith-based networks was a barrier to sustaining their enthusiasm to do CE. While some refreshments and mementos were offered to acknowledge their social mobilization efforts, most felt that these were not enough.

“*These felicitations* (providing certificates during a village gathering) *were given to the local headman for more than 90% vaccination coverage*, *acknowledging their contribution*. *But there was no monetary incentive*.*”*

### Interpersonal-level CE facilitators

Participants described an evolution in messaging–previously, authoritative messaging indicated that vaccination was a parental onus, with phrases like, *“vaccinate your child*,*” “don’t forget vaccination your baby must get*,*”* and *“Be wise*, *fully immunize your child*,*”* published in the National Health Portal and all the community-facing SBCC materials. The current messaging tagline in Hindi language, “*5 saal 7 baar*, *Choote na teeka ek bhi baar”* instead depicts the importance of routine immunization with the message that a child should be immunized seven times in first five years of its life, reflecting the Ministry’s salutogenic model clarifying the difference between vaccination and immunization especially at a time when communities might be overwhelmed with introduction of several new vaccines under the Ministry’s immunization program and that paternalistic vaccination messaging could rather plummet vaccination rates than otherwise.

Another participant explained the role of visuals, such as pictures, or drawings, markedly increasing communities’ attention to and recall of health education. Citing the example of the Mission Indradhanush logo with “*the rainbow*, *an umbrella of seven colors*,*”* he explained that it indicated vaccination for seven vaccine preventable diseases—diphtheria, whooping cough, tetanus, polio, tuberculosis, measles, and Hepatitis B—available at the community outlet or provided by the community-level frontline healthcare workers seven days a week. Others reflected on the use of social media to provide information neutrally, positively, and without blame:

“*Like today there is a social media strategy after M-R (Measles-Rubella) campaign*. *Health Ministry is using its Twitter account and putting in people to tweet*. *What has Twitter to do with Health Ministry*? *Yes*, *it is becoming important*. *(Decisionmakers are thinking) How to send messages through WhatsApp so that the messages reach*? *Earlier messages were through texts*. *Who is there to read your texts*? *Who is there to forward your texts*? *Nobody is interested*. *So*, *messaging is getting more and more creative*.*”*

Especially, in the context of the predominantly patriarchal society in India, interpersonal efforts by healthcare workers to sensitize mothers-in-law and husbands to “allow” mothers to vaccinate their children was also considered a CE enabler:

“…*mostly the men would talk and they will not listen*. *So*, *we went to the barber’s shops*. *That was the only place where men used to be silent during their haircut*. (Our Communication personnel) *gave some danglers and aprons with messages for immunization* (SBCC materials) *to the barbers and their associations*. *And then we taught them (barbers) to talk to their customers around immunization of the latter’s child*.*”*

### Interpersonal-level CE barriers

Study participants indicated that training of trainers for the ASHAs, ANMs and AWWs, sponsored by the MoHFW and UNICEF, was a CE facilitator because it trained these frontline healthcare providers on interpersonal communication skills. However, time constraints for these trainings were highlighted as a barrier by some study participants:

“*If you look into* Boosting Routine Immunization Demand Generation (*BRIDGE) training*, *you are building somebody’s interpersonal communication capacities*. *Private companies invest huge amount(s) where they train their marketing personnel how to go and talk to somebody*. *Whether I am going to talk to the business executive or whether I am going to sell from door to door*, *that engagement strategy is a very critical and we need to invest more time and energy for that*.*”*

One participant also noted that family-centric materials had not been developed for certain sub-populations:

“Wives of men based in the Middle-East often did not get a timely affirmative vaccination decision (from their husbands), mostly leaving their children unvaccinated. We need to develop some strategy for this group.”

This lack of tailoring was also identified in vaccine policy documents. For example, both the FAQ on Immunization for Religious Leaders, Media Persons, Civil Society Organizations, Influencers & Other Stakeholders, 2017, and the FAQ on Immunization for Parents & Caregivers, 2017, though designed for separate stakeholders, contained similar content.

Finally, in contrast to the enthusiasm of the study respondents about using social media for vaccine messaging, none of the policy documents highlighted any social media proliferation in rural and semi-urban India or scaling up the social media strategy among clusters of communities where vaccination-related conservative values are widely shared.

### Individual-level CE facilitators

Several participants recommended institutionalized support and participatory CE, but no one was able to provide examples of decision-making entities engaging with communities at an individual-level except home visits for polio vaccination and investigation of serious cases post-vaccination. These instances typically were tied to the polio eradication campaign, or adverse events following immunization, in general. Since, by design, the polio eradication campaign was a house-to-house delivery program, and adverse event cases mandated an individual-level inquiry, some participants did not categorize these as individual-level CE enablers. Only one participant cited “*going an extra bit*” in an individual capacity, which might be considered close to an individual-level facilitator. However, it was for vaccination uptake rather than CE:

“…*delivering vaccines to the last mile is one of the major challenges*. *There were two places where the vaccine was transported through helicopter*. *First time Government of India gave that fund*. *Although there were only 15 beneficiary children but I personally [from UNICEF] requested the Government that if you say that 100% children are to be immunized*, *somehow you have to send the vaccines to that place*.*”*

### Individual-level CE barriers

To some degree, participants expressed top-down interpretations of individual-level vaccination support. One decisionmaker shared an interesting example of hiring a *“muscle-man” in one of the states in Northern India* to “*convince the communities to vaccinate their children*.*”* Interestingly, participants positioned this individual-level “coaxing” as the only way to counter the demand-side barriers to CE, variously listed as: communities’ vaccine resistance, ignorance, lack of literacy, misinformation, confusion between vaccination and immunization, logistics (remembering to get the vaccine), and relationships with the local health provider. One participant said:

“*I would like to talk about the people who are not coming forward*. *This 10% population are the ones who are resisting*. *That is the population that needs to be taken care of*, *reached*, *or taken out of their homes to reach the immunization sessions*.*”*

## Summary of the participants’ follow-up convening meeting

A full-day convening meeting of all the participants was held in New Delhi on December 19, 2018. Three participants could not make it to this meeting and met the first author (TD) later in January 2019. These meetings provided the opportunity for a review of observations and verification of results. Participants agreed with the reported results and recommended a comprehensive and salutogenic operational definition of CE for effective vaccine introduction and uptake.

“*CE is an upstream policy imperative*, *rather than downstream interventions*, *to build trustworthy relationships between vaccine decisionmakers and communities*. *It involves demystifying science and transparent communication for empowered community agency*. *This [CE] would enable communities to critically analyze vaccine related myths and misinformation and enable knowledge co-production in building community-sensitive vaccine policies and programs*. *CE is incumbent to sustained political-will and resources to ensure evidence-informed*, *tailored*, *vaccine policies and programs*, *providing equitable*, *quality*, *and tangible vaccination and capacity building benefits to community members*.*”*

## Discussion

Using SEM in this study helped not just to identify and categorize the ‘enablers’ and ‘barriers’ of CE in vaccination in India, but also to challenge the rather romanticized notion of CE. This ecological conceptual framework allowed the identification of the factors more freely and contextually, without the constraints of a rigid theoretical framework. Operationalization of the CE concept by the national level vaccine decisionmakers was unique and significant to policy scholarship, and implementation research, wherein a participatory discursive processes led to the broader framing of ‘CE in vaccination’ as: (1) a cardinal policy imperative and social determinant of health for vaccination equity, and (2) a civic engagement process [61], leading to social capital and trust building between communities, vaccine science and vaccine decisionmakers, for vaccination uptake [62].

While recent studies exploring CE in the field of vaccination and vaccine research have highlighted pertinent links between CE and vaccination uptake rates [63, 64]. This study goes another step further, to identify the actual implementation and management-level ‘factors’ which elite decisionmakers believed and experienced in doing CE, and the ways in which they perceived these factors are linked to vaccine acceptance and vaccination uptake. It also identified vaccine specific community contestations [HPV vaccines], which signals for differential CE strategies for childhood and adolescent/adult vaccinations [65].

Despite the overarching support for CE by vaccine decisionmakers and policy documents in India, its conceptualization, facilitation, and implementation in different scenarios—for sub-populations, or during outbreak versus non-outbreak times—remain elusive. Vaccine decisionmakers appreciated the study’s timeliness, both retrospectively and prospectively, as an effort both to understand past CE challenges and successes and to appropriately plan for future CE interventions, which certainly contributed to community empowerment discourse and systems discourse related to CE.

That said, due to ongoing political activism and conflicts and community backlash, decisionmakers were cautious, which posed challenges during the data collection stages, even in getting entry to the study participants’ offices. This issue remains highly charged, especially in certain topical areas. For example, the MoHFW shelved the introduction of the HPV vaccine after representatives of a political party urged the Prime Minister not to introduce the vaccine because it ‘*brings ignominy to the scientific community in the country and sells the country to vested interests’* [66], while, at the same time subsidized food under the Public Distribution System of the Government of India were denied to families until they vaccinated their children [67]. Fortunately, the researcher’s prior knowledge of the sector and familiarity with the decisionmakers facilitated access [68], rendering this dataset unique.

While the Rwanda and Bangladesh studies that were cited earlier provided guidance in designing the tools of this study [69, 70], these studies have characterized CE within a limited range: tailoring vaccination strategies to the local context, engaging community health workers and local stakeholders for decentralized vaccine outreach, and community meetings to deliver information about vaccination. A potential limitation of such approaches is that it reduces the community’s identity to “vaccine recipients” with little or no agency to invigorate their social desire for pharmacovigilance [71]. Prior research has also identified an overarching utilitarian conceptualization of CE by decisionmakers [72], which may conflict with decisionmakers’ stated desire for empowered and discerning communities who demand vaccines. Based on this study, we argue that this might not reflect a lack of effort by the vaccine decisionmakers to address CE priorities. Rather, it could be function of the complexities involved in standardizing CE strategies for a diverse country like India, which has the world’s largest annual cohort of 27 million vaccine eligible children.

CE barriers identified in this study, such as the lack of any CE-specific indicators or strategy documents, the absence of dedicated CE staff, and a culture of silence for CE strategies for contested vaccines wherein several states of India demanded stalling the HPV and M-R vaccines [73, 74], suggested revisiting the traditional social mobilization approaches. Some existing strategies perceived to be CE may inadvertently reinforce existing power differentials, exacerbated by the community’s poverty and livelihood crises, negative beliefs about vaccinations, and poor treatment of communities by healthcare workers and authorities [75–77]. Poorer, rural, minority communities, and women, were presented as the most suspicious and resentful of vaccines and government authorities and were collectively identified as ‘hard to reach populations.’ Prior research might suggest that this was driven by incidents of ‘covert resistance by communities’ [78], ‘reactance’ among communities to regain their freedom of choice [79], and suspicions about ‘sudden interventions’ by the authorities [80–83].

While most decisionmakers and policy documents in this study dismissed communities’ vaccine resistance as *“not vaccine-related*,*”* vaccine communication needs to frame and address the social construct of vaccines and vaccinations per se in the caste, class, and patriarchal context of India. Often, it was portrayed that a community’s disapproval of government policies or paternalistic healthcare providers was driven by upper class and caste-group people or men, while the poor, minorities, and women in those same communities were perceived [by decisionmakers] as being vaccine hesitant and resistant. While further studying is needed, this may have implications for the appropriate targeting of CE efforts and the content of those efforts [84]. Further, intersectionalities and predicaments among the socio-economically disadvantaged are important to consider, as these communities try to negotiate between social pressure and preventive health, especially because subsistence typically takes priority over health in general, and immunization in particular, among these populations [85].

Based on the information discussed regarding vaccine gatekeepers, it seems that strategic CE with those gatekeepers might yield positive results, because this would not only enable access to communities via these gatekeepers but could also unfurl socio-cultural sensitivities in SBCC materials when transacted in local dialects, while also empowering communities to have meaningful discussions between vaccine supporters and gatekeepers [86]. This would need to be studied more closely, but as we highlight in a related paper [87], strategic engagement with vaccine gatekeepers is important because some decisionmakers indicated that they did not have a strategy to work with gatekeepers, who were variously described as people who resisted a particular vaccine or vaccination, including *‘activists’*, especially those *‘associated with any anti-vaccine political groups’* [88].

There was an overarching recognition that successful CE requires demystification of vaccine-related capacity building among stakeholders and information sharing among communities, which resonates with findings from prior studies [89, 90]. However, Kilpatrick (2009) expressed concern that the biomedical training model can impede participatory approaches because trainees might be attracted to utilitarian gains rather than empowerment approaches of CE [91]. At the same time CE appears to be an important part of understanding and developing communities’ understanding of the science of vaccines. This may include understanding how vaccination works in general [92], the concept of herd immunity [93], and the implications of immunization for babies who are too young to be vaccinated or immuno-compromised children, who are the first potential victims of low vaccination rates [94].

### Limitations

This study encountered methodological challenges associated with the elite interviewing approach, especially when the policy processes being studied played out in real time. There was a risk that the data being provided were limited (obviously so, in some cases–as noted in the Results) due to political needs. Further, the factors affecting CE in a specific setting might not be interpreted correctly based on information from national-level decisionmakers and policy documents. To address this, the first author regularly summarized and fed back interpretations to the participants for respondents’ validation. This was also managed by the convening of a final meeting wherein stakeholders could review the results of the study.

## Conclusion

While there appears to be a considerable amount of political will to support CE, the precise ways in which it can be implemented, and how it can be judged effective, are not well studied or documented. As a result, one key recommendation from this study is to ensure that data collection systems capture CE data. For example, immunization program evaluations should consider including CE needs assessments, carrying out process documentation (story arc of CE) and conducting formative evaluations of CE outcomes at all the social ecological levels. Future CE barrier studies should identify key outcome indicators of CE based on a critical awareness of the history and nature of evolving relations between communities, vaccines, and vaccine-providing authorities in diverse cultural, economic, disease outbreak, and political contexts.

## Supporting information

S1 File (DOCX)
