# Supplementary material for: Perceived enablers and barriers of community engagement for vaccination in India: Using socioecological analysis
Source: PLoS One. 2021 Jun 25;16(6):e0253318. doi: 10.1371/journal.pone.0253318 (PMC8232440; doi:10.1371/journal.pone.0253318)
Supplement: S1 File — (DOCX) [file pone.0253318.s001.docx]

S1 File

Perceived enablers and barriers of community engagement for vaccination in India: Using socioecological analysis

Email to study participants

Name

Title

Address

Via email

RE: Interview request for a study about community engagement in vaccine planning, policy, and rollout

Dear Title [name],

I write to invite you to participate in a brief [30 minutes] interview to discuss your institution/agency’s experience with and engagement of communities in vaccine policy and planning. This interview is part of my doctoral dissertation research on ‘Decision makers’ Ownership and Support of Community Engagement to Improve Adoption and Uptake of New and Emerging Vaccines in India’. I have enclosed study information for your consideration. Dr. Beth Meyerson and Dr. Priscilla A. Barnes are the co-chairs of my dissertation committee.

India has made tremendous progress during our “Decade of Vaccines” (2010-2020) by introducing multiple new vaccines along with increasing access to new and underused vaccines. Despite our progress, vaccine uptake is less than it should be. Understanding community engagement in vaccine policy and planning may help to identify opportunities to further advance our vaccine efforts.

As a citizen of India, and a committed socio-behavioral scientist in community health, I have worked with Indian non-profit organizations for 15 years on issues of HIV and cervical cancer prevention as well as promoting use of prevention tools among the rural and vulnerable sub-populations of women and girls.

You are invited to participate in an interview because of your commendable vision and advocacy for vaccines in India. If you agree to participate in this study, please reply to this email and indicate your availability (dates/times) during this period: December 6, 2017- January 26, 2018. I will be in India during this time period, but can also arrange for an online/video interview if this is better for you.

I am hopeful for your decision to participate in a brief interview, and look forward to sharing the results of the study with you.

Sincerely

Tapati Dutta

Doctoral Candidate

Research Associate, Rural Center for AIDS/STD Prevention

Indiana University School of Public Health-Bloomington
